# Secondary Hip Osteoarthritis due to Neurofibroma Treated with Total Hip Replacement

**DOI:** 10.1155/2012/173921

**Published:** 2012-10-23

**Authors:** Suksan Tangsataporn, Alireza Shakib, Paul R. Kuzyk, David J. Backstein, Allan E. Gross, Oleg A. Safir

**Affiliations:** Division of Arthroplasty, Orthopedic Department, Mount Sinai Hospital, 600 University Avenue, Suite 476A, Toronto, ON, Canada M5G 1X5

## Abstract

*Background*. Local plexiform neurofibroma can lead to deformity of the pelvis, valgus deformity of femoral neck, and joint capsule laxity. We report a case of secondary hip osteoarthritis with subluxation and coxa vara deformity resulting from an extra-articular neurofibroma treated with total hip replacement. *Case Description*. A 39-year-old man had a large benign plexiform neurofibroma at buttock which induced secondary osteoarthritis of the hip. Conservative treatment of tumor was selected because the patient had low chance of malignant transformation due to absence of other neurofibromatosis features. However, due to secondary osteoarthritis he underwent total hip arthroplasty. Anterior capsulotomy was selected to avoid large posterior hip tumor mass. In order to avoid the difficulties associated with setting tension of the abductor muscle, modified trochanteric slide osteotomy with trochanteric advancement, lateralized cup placement, and extended neck offset were used. One year after the surgery, the patient had excellent clinical function, hip stability, leg length equality and was satisfied with the outcome. *Clinical Relevance*. We concluded that the modified trochanteric slide osteotomy with trochanteric advancement represents a valuable approach for THR in patients with extremely elongation of the hip abductor and secondary hip osteoarthritis resulting from extra-articular neurofibroma.

## 1. Introduction

 By definition, plexiform neurofibromas are tumors of the peripheral nerve sheath that form along large nerve trunks, multiple small branches of nerves, or spinal roots (nerves that arise from the spinal cord) causing enlargement. Neurofibromatous tumor can cause bone erosion due to compression of the adjacent bone [1, 2]. Furthermore, local neurofibromas can lead to deformity of the pelvis, valgus deformity, and joint capsule laxity, all of which predispose a person to dislocation [3]. Recurrent dislocation leads to subsequent development of progressive hip arthritis [4]. Therefore, surgical approach, offset, soft tissue tension, and stability are particular challenges when performing THR in this population of patients.

 We report a case of secondary hip osteoarthritis with subluxation and coxa vara deformity resulting from a large extra-articular neurofibroma in a 39-year-old man. The aim of this paper is to emphasize the surgical technique in order to avoid early and late postoperative complications. 

## 2. Case Report

A 39-year-old accountant was evaluated by the orthopaedic oncology service at our institution. The patient presented with a 9-year of right hip pain. He noticed a swelling in his right buttock approximately 6 years ago that was growing for 3 years. At the time of presentation, the pain was localized to the right groin and did not radiate. However, he was unable to walk more than 30 min. Pain was worse during vigorous activities and he was unable to participate in sports. The patient walked with a significant limp. 

 On examination, a large soft tissue mass was palpated in his right gluteal region. The mass was soft, not tender and mobile with no overlying skin change. Right hip motion included flexion of 100 degrees, fixed flexion deformity of about 15 degrees, and abduction and adduction of 30 degrees. Both external and internal rotations were limited on the right side to 15 degrees. Pain was present with all planes of motion. He walked with a significant lurch and his right leg was 5 mm shorter than his left. Neurological examination revealed no abnormal findings.

**Figure 1:**
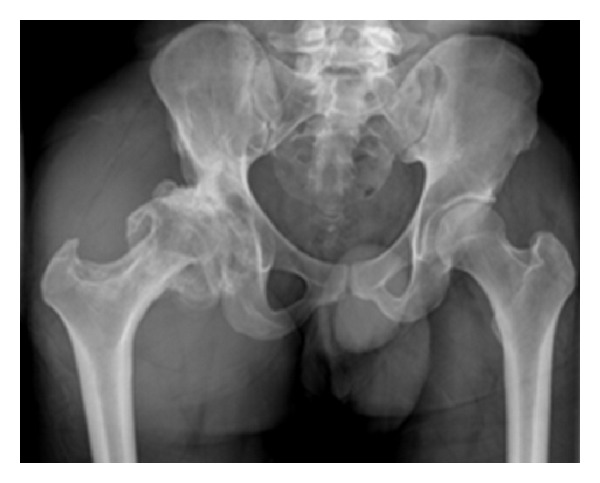
The preoperative radiographic demonstrates lateralization and subluxation of the right hip, osteoarthritis of hip joint, and excessive offset deformity with a longer neck of femur.

**Figure 2:**
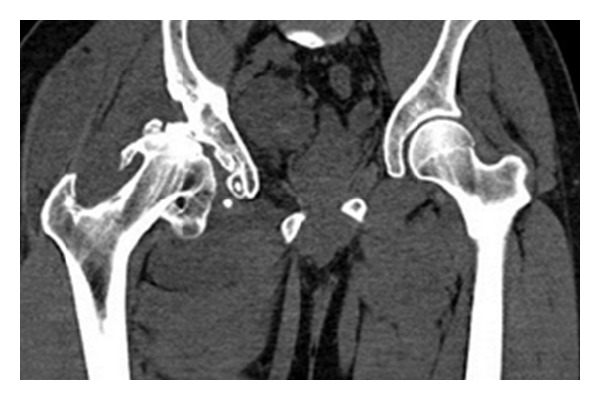
The CT scan coronal cut demonstrates numerous osteophytes around the femoral head and acetabulum.

Radiographs of the right hip showed significant lateralization and subluxation with change of osteoarthritis, coxa vara deformity, and a long neck of femur (Figure 1). CT scan confirmed the presence of subluxation of the right hip with numerous osteophytes around the femoral head and acetabulum (Figure 2). 

**Figure 3:**
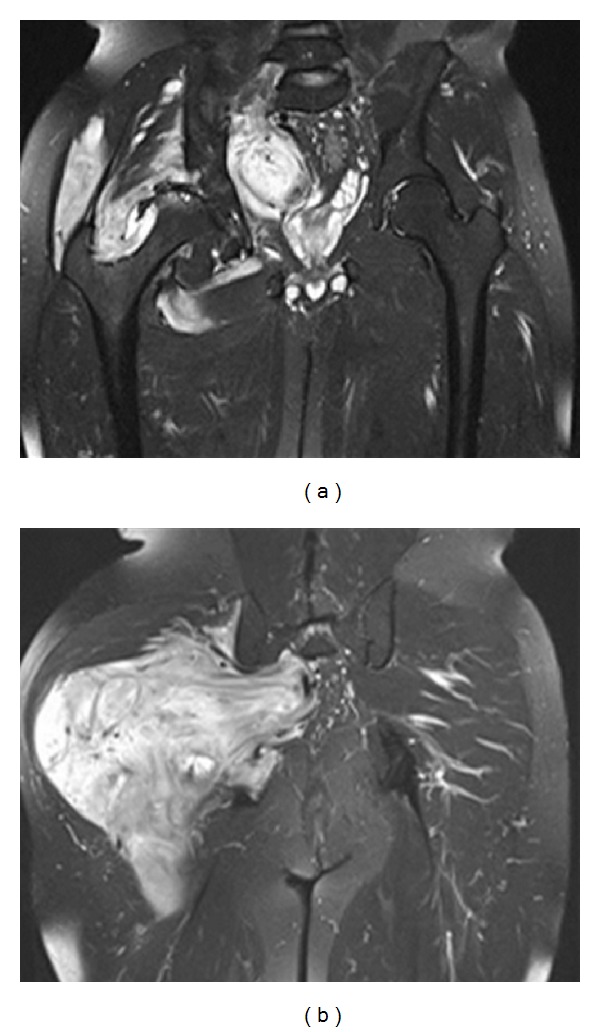
(a) The coronal T2-weighted demonstrates the vascular enhancing mass directly abuts the synovium of the right hip joint and wraps around the femoral neck. (b) The coronal T2-weighted demonstrates a large massive vascular enhancing mass which largely is posterior to the right hip.

 MRI revealed a large massive vascular enhancing mass of 23 × 13 cm which largely was posterior to the right hip and extended above the femoral neck. The mass extended through the neural foramina at the L5-S1 level (Figures 3(a) and 3(b)). 

 Ultrasound-guided biopsy of the area was performed in order to rule out malignancy. Histological examination of the tissue revealed plexiform neuro fibroma. Aspiration was performed and infection was ruled out. Since the patient did not have any other features suggestive of neurofibromatosis, he had a very low chance of malignant transformation. Due to large size and pelvic location of the tumor, the orthopaedic-oncologist recommended conservative treatment. The patient's pain was mainly experienced in the groin pain and not the mass itself. The patient was ultimately referred to our arthroplasty unit for consideration of total hip arthroplasty. Due to the secondary arthritic change which had developed, it was elected to proceed with total hip arthroplasty.

 The operation was performed utilizing a modified trochanteric slide osteotomy approach. Anterior capsulotomy was chosen to avoid posterior hip tumor mass. There was a large posterior tumor in the buttock under the tensor fascia lata and a small mass at the inferior and posterior aspect of the acetabulum. An outrigger (Stryker company), a device for measuring leg length and offset, that placed on the iliac crest was used. The femoral neck was carefully cut at a length base on preoperative templating. Because it was quite difficult to remove the deformed femoral head, the femoral head had to be split in half *in situ* to remove from the acetabulum. The acetabulum had a deficient anterior wall. Elongation of the gluteus medius tendon and muscle was shown. In order to improve tension of hip abductor, minimal reaming was performed to avoid deep position of the cup. A trabecular metal shell (Zimmer, Warsaw, IN, USA) size 58 mm was impacted and liner was positioned into the cup. A large diameter head (36 mm) was used to reduce the risk of dislocation. 

 The M-L taper, extended neck offset, femoral component (Zimmer, Warsaw, IN, USA) was impacted and the head was attached. The hip was reduced and the outrigger was used again to check the offset and length. The stability and range of motion were tested. The trochanter was reattached on the lateral femoral cortex distal to the original bed in order to increase the abductor tension and stability of hip joint. 

**Figure 4:**
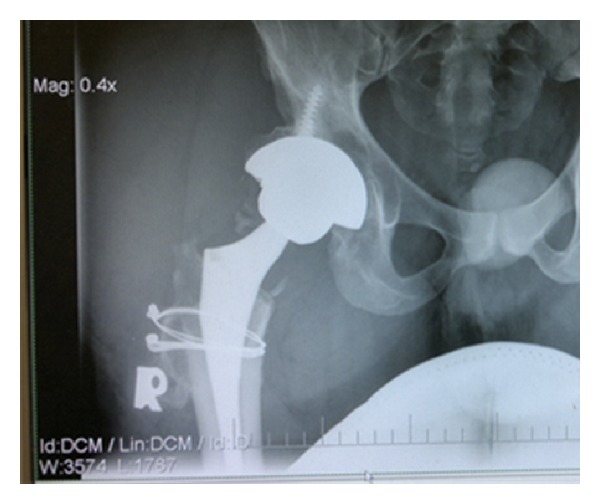
The anteroposterior radiograph of the right hip at 1 year postoperative: showing relative lateral position of the socket to improve offset and 4-5 cm distal advancement of the greater trochanter.

 The patient was rehabilitated weight bearing as tolerated and used an abduction brace for 6 weeks. On physical examination at the fourth month postoperatively, the patient demonstrated that he had normal gait, equal leg lengths, and a good range of motion of the right hip with flexion to 90 degrees, abduction to 45, adduction to 30, and external rotation to 30 degrees. At 1-year followup, the patient continued to do extremely well without pain, normal gait, and a good range of motion. On examination, he walked without a Trendelenburg lurch. At this followup, he had a Harris hip score of 93 points. The anteroposterior radiograph of the right hip showed relative lateral position of the socket to improve offset and distal advancement of the greater trochanter (Figure 4).

## 3. Discussion

Plexiform neurofibromas are most often congenital tumors associated with neurofibromatosis type 1 (NF1) that can cause accelerating bone and soft tissue growth [5]. Although these tumors are usually benign, there is a 2–5% chance of malignant transformation in the setting of NF1 [6]. In this case, the neurofibroma was felt to have a low chance of malignant transformation but was clinically asymptomatic. However, decisions about surgical treatment must be made judiciously and individualized for each patient [5].

Neurofibromas distant from the hip joint have been hypothesised to cause pathological dislocation. Mechanical instability due to weakness of the abductor muscles caused by spinal-cord tumor and tumor impingement which have been suggested as a predisposing factors [3, 7]. In the case of this patient, we found no tumor in the spinal cord and no other signs of neurofibromatosis or abnormal neurological examination. Although the X-ray shows the same shallow acetabular at the right hip as in dysplastic hip, coxa vara, long neck of femur, and normal size of femoral sharp are not the common deformity in dysplastic hip. His hip subluxation was a result of the large neurofibroma putting pressure on the hip joint and subsequently he developed progressive arthritis. 

 If adjustments in modular implants, such as offset acetabular liners, offset femoral stems, and longer neck lengths do not provide stability without excessive lengthening (>1.5 to 2.0 cm), a trochanteric slide osteotomy can be performed with distal advancement [8], for our patient, because his neck of femur was very long and his hip was in varus deformity. Moreover, Lateral subluxation was present. Therefore, severe lax abductor mechanism was concerned before surgery. We used lateral position of the cup and an extended neck offset of femoral component to improve tension of hip abductor. However, they cannot provide enough hip stability. The trochanter was then advanced 40–50 mm distally to the previous level and reattached to the lateral cortex of femur which increased the abductor tension (Figure 4). There were few studies [9, 10] that reported an average 16–18 mm (range 10–30 mm) of distally trochanteric advancement for treatment of recurrent dislocation after THR. When compared advancement with other study, our patient needed much more distally trochanteric advancement to provide enough stability after THR. Therefore, trochanteric advancement is an ideal method that can improve hip stability after THR in extreme elongation of hip abductor without excessive leg lengthening. 

 Another advantage of using trochanteric slide osteotomy approach is to preserve the vastus lateralis muscle origin and decrease the incidence of trochanteric migration [5]. Anterior capsulotomy was performed with this approach to avoid large posterior hip tumor. A large femoral head (36 mm) was chosen to reduce dislocation risk. The outrigger device was used to check the offset and leg length. If the hip joint is still unstable after all techniques are made use of, a constrained liner is an option.
